# Understanding discharge communication behaviours in a pediatric emergency care context: a mixed methods observation study protocol

**DOI:** 10.1186/s12913-017-2204-5

**Published:** 2017-04-17

**Authors:** Janet A. Curran, Andrea Bishop, Amy Plint, Shannon MacPhee, Roger Zemek, Jill Chorney, Mona Jabbour, Stephen Porter, Scott Sawyer

**Affiliations:** 1 School of Nursing, Dalhousie University, Halifax, Nova Scotia B3H 4R2 Canada; 2 Department of Emergency Medicine, Children’s Hospital of Eastern Ontario, Ottawa, Ontario K1H 8M5 Canada; 3 Department of Emergency Medicine, IWK Health Centre, Halifax, Nova Scotia B3H 4R2 Canada; 4 Department of Anesthesia, Faculty of Medicine, Dalhousie University, Halifax, Nova Scotia B3H 4R2 Canada; 5 Division Of Emergency Medicine, Hospital for Sick Children, Toronto, M5G 1X8 Ontario Canada; 6 Pediatric Emergency Medicine, Manitoba Children’s Hospital, Winnipeg, Manitoba R3A 1S1 Canada; 7 Children’s Hospital of Eastern Ontario Research Insititute, Ottawa, Ontario Canada

**Keywords:** Mixed Methods, Video observation, Transitions in Care, Pediatric Emergency Medicine, Communication behaviours

## Abstract

**Background:**

One of the most important transitions in the continuum of care for children is discharge to home. Optimal discharge communication between healthcare providers and caregivers (e.g., parents or other guardians) who present to the emergency department (ED) with their children is not well understood. The lack of policies and considerable variation in practice regarding discharge communication in pediatric EDs pose a quality and safety risk for children and their parents.

**Methods:**

The aim of this mixed methods study is to better understand the process and structure of discharge communication in a pediatric ED context to contribute to the design and development of discharge communication interventions. We will use surveys, administrative data and real-time video observation to characterize discharge communication for six common illness presentations in a pediatric ED: (1) asthma, (2) bronchiolitis, (3) abdominal pain, (4) fever, (5) diarrhea and vomiting, and (6) minor head injury. Participants will be recruited from one of two urban pediatric EDs in Canada. Video recordings will be analyzed using Observer XT. We will use logistic regression to identify potential demographic and visit characteristic cofounders and multivariate logistic regression to examine association between verbal and non-verbal behaviours and parent recall and comprehension.

**Discussion:**

Video recording of discharge communication will provide an opportunity to capture important data such as temporality, sequence and non-verbal behaviours that might influence the communication process. Given the importance of better characterizing discharge communication to identify potential barriers and enablers, we anticipate that the findings from this study will contribute to the development of more effective discharge communication policies and interventions.

## Background

One of the most important transitions in the continuum of care for children is discharge to home. This is especially true in a pediatric emergency department (ED) context where there has been a recent call to action for development of quality and safety indicators for practices such as discharge communication [1, 2]. In 2007, the US Joint Commission identified communication as the most important factor associated with sentinel events in a root cause analysis. Communication continues to be identified as one of the top 10 risk factors impacting quality care. Optimal discharge communication between healthcare providers and caregivers (e.g., parents or other guardians) who present to the ED with their children is not well understood. Current research literature regarding discharge communication interventions in the context of pediatric emergency care is equivocal and predominantly focused on evaluating different delivery formats (e.g. handouts, videos) or strategies to encourage adherence of the recipients of the information (e.g. parents) with little attention given to communication behaviours or the context in which the communication occurs [3]. The lack of policies and considerable variation in practice regarding discharge communication in pediatric EDs may pose a quality and safety risk for children and their parents [4]. This gap in practice requires urgent attention given the volume of visits to an ED by children with the majority (87%) being discharged to home under the care of their parent/caregivers [5, 6].

Discharge from an ED can be a time of significant stress for caregivers and they should leave with the necessary knowledge and skills to effectively manage a child’s care at home. The discharge process should communicate important information, verify comprehension, and tailor the discharge instruction to address areas of misunderstanding [7]. Poor quality ED discharge communication has been found to impact health outcomes, parent satisfaction with care and health care utilization (e.g. unscheduled return visits to the ED) [8, 9]. Comprehension of discharge communication has been shown to be an important factor in promoting adherence to discharge instructions and preventing unnecessary return visits; however, comprehension is rarely assessed in practice [10]. Following discharge from the ED, patients are often unable to cite their diagnosis, list medications they received, outline post-ED care, or identify when to seek further medical attention [11–13]. Health literacy and numeracy of caregivers also influence discharge communication but are rarely assessed in practice [14–16].

Discharge communication process and interventions vary within and between EDs [17, 18]. Unique characteristics of the ED setting, such as frequent interruptions to providers’ workflow and task completion, can complicate effective communication of important information and the time intended for discharge communication can sometimes be compressed to address other, more urgent, activities [19, 20]. Furthermore, younger children and patients who visit during the busiest hours are more likely to return to the ED [21]. Caregivers may be anxious about their child’s health status and their ability to manage at home, which may influence their readiness to learn or acquire the necessary skills. This can be compounded if parents/caregivers feel that their child is not ready for discharge, or feel incapable of caring for the child at home [22].

While chart audits and parent recall studies provide a snapshot of discharge communication, they are limited by potential missing information and reliance on recall and fail to fully capture the complexity of the discharge communication process as it occurs in a pediatric ED. Observational studies can increase our understanding of complex problems and assist with generating hypotheses for future testing in rigorous evaluations [23]. However, observations in clinical practice can pose a challenge for researchers to capture all of the details relevant to a phenomena [24]. Video recording of the discharge communication process as it occurs in real time can produce a rich data set of both verbal and non-verbal communication behaviours, preserving the temporal and sequential structure that is critical for characterizing the interaction. To date, there are no video observation studies reported in the pediatric ED literature that characterize discharge communication with caregivers. This study is a critical step in identifying clear targets for designing effective discharge communication interventions.

### Objectives

The aim of this mixed methods study is to better understand the process and structure of discharge communication in a pediatric ED context. Specific study objectives include:

1. To describe content and process elements that are included in discharge communication across different illness presentations in a pediatric ED
2. To describe common verbal and non-verbal behaviours of caregivers and healthcare providers during discharge communication
3. To determine the association between content and process elements and caregiver recall and comprehension of discharge communication
4. To determine the association between verbal and non-verbal behaviours and caregiver recall and comprehension of discharge communication

## Methods/Design

This exploratory observational study uses a mixed methods approach to capture multi-level data (patient, caregiver, healthcare provider) relevant to discharge communication for six common illness presentations in a pediatric ED: (1) asthma, (2) bronchiolitis, (3) abdominal pain, (4) fever, (5) diarrhea and vomiting, and (6) minor head injury. These six presentations were chosen to provide a mix of both chronic and acute illness presentations and to ensure a robust sample size. Using multiple data sources to develop an understanding of phenomena can neutralize biases or clarify misconceptions that may be formed when only using one source or method [25]. This study was peer-reviewed and funded through a Nova Scotia-Canadian Institutes of Health Research (CIHR) Regional Partnership Program (RPP) grant. The protocol and study has been approved by Pediatric Emergency Research Canada (PERC) and the Research Ethics Boards of both participating EDs (protocol version 2016.2.2).

### Study population

This study includes children, caregivers, and ED healthcare providers and trainees in two urban academic pediatric EDs in Canada.

### Caregivers

We will recruit caregivers (e.g., legal guardians) of children who present to a pediatric ED with one of the six diagnoses of interest. We aim to recruit 8-10 child/caregiver dyads per illness at each site (total 96-120 child/caregiver dyads), resulting in a total of 16–20 cases for each illness presentation. As Yin [26] notes, when examining a phenomenon in its real-life context, there will always be too many “variables” for the number of observations. Consequently, standard observational design criteria are not relevant. The development of multiple cases for each illness presentation will allow us to compare and contrast differences within and between cases [26]. Criteria for recruitment includes: children 0–16 years of age accompanied by a legal guardian who present with one of the six illness presentations and a Canadian Triage and Acuity Scale (CTAS) score of 3, 4 or 5 [27]. Children who arrive by ambulance, are unaccompanied, have a CTAS score of 1, are admitted to hospital, have previously been enrolled in this study or are non-English speaking will be excluded. Caregivers will be approached for consent after the triage nurse in the ED has assessed their child.

### Healthcare providers

All ED physicians, nursing staff, trainees/interns/residents/fellows and other learners, health care aides, and allied health care providers will be eligible to participate in this study.

### Design

Non-participant observation using video recording of clinician-caregiver communications in the ED will be combined with chart audits and follow-up telephone interviews with the caregiver to examine the practice of discharge communication. Two small video cameras with a wide-angle lens and a high-fidelity microphone will be installed in opposite ceiling corners of a patient assessment room in the ED to facilitate recording of the encounter. Although previous studies suggest video and audio recording is unlikely to influence provider-caregiver communication [28–30], this data collection strategy is novel for examining communication in a pediatric ED context. Therefore, we will minimize the potential impact that the visibility of the video equipment may have by encasing the video cameras under an opaque dome cover and placing them in discrete locations in the patient room. A Research Assistant (RA) will control the cameras remotely using video recording software on a laptop from his/her post outside of the patient’s room. A purposive, maximum variation sampling strategy will be used to ensure diversity across four parameters: (1) illness presentation, (2) health care provider, (3) day of the week, and (4) time of day. All study participants will be consented prior to recording.

#### Caregiver measures

A caregiver demographic form will capture information regarding age, gender, highest level of education, number of children and caregivers in the home and information about previous visits to the ED. If a child presents with more than one caregiver, the family will be asked to identify the primary caregiver for study participation. Caregiver stress will be measured using the State-Trait Anxiety Inventory (STAI) scale, a 20-item self-report survey pertaining to anxiety, and a 10-point visual analog scale [31]. The Medical Term Recognition Test (METER) will be administered to assess caregiver health literacy [32]. Caregivers will be asked to complete all three measures in the waiting room after consent to participate in the study has been established. The visual analog scale will be repeated in the ED after the child has been discharged to note any changes in caregiver stress before and after the visit.

#### Non-participant observation

A video recording device will be placed in two assessment rooms in the ED. The RA will act as a non-participant observer during the patient encounter sitting outside of the room and not readily visible to the provider or caregiver. Field notes will be collected electronically during the period of observation to help illuminate the context and conditions under which discharge communication occurred. This will include any distractions or events that take place in the hallway outside the child’s room or in the department that might interfere with communication. Video recording of all verbal and non-verbal communication between consenting clinicians and caregivers will begin the moment the child is placed in the assessment room. The RA will be responsible for activating the video recorder every time a clinician enters the room. Caregiver and patient communication will not be recorded unless a healthcare provider is present in the room. Recording of all provider/caregiver interaction will continue until the patient is discharged home.

#### Chart audit

Standardized data abstraction forms will be used to collect information from the patient's chart, including age, gender, presenting diagnosis, procedures, diagnostic tests or medications administered in the ED and discharge instructions.

#### Caregiver follow-up survey

At time of consent, an RA will seek permission to contact caregivers to complete a brief email or telephone follow-up survey within 72 h of discharge. The caregiver’s email address and telephone number and the most convenient day and time to call will be collected. Those participants who indicated a preference for email follow up will receive an email with a link to the survey within 72 h. If the caregiver does not complete the survey within the 72 h time frame, the RA will contact the caregiver to complete the survey over the telephone using the contact number provided. The follow-up survey will measure recall and comprehension of the information provided during their ED visit regarding their child’s diagnosis, diagnostic procedures performed in the ED, medications administered, and the five discharge messages identified as essential for their illness presentation previously reported in our Delphi study [33]. Caregivers will also be asked about their confidence in their ability to follow the instructions at home and their overall perceptions of the discharge communication process.

#### Department census data

Total 24 h ED census data will be collected for each observation day. This will be used as a measure of patient load and department busyness on data collection days and will assist with providing context for understanding discharge communication behaviours.

#### Data management

All data will be managed using a Research Electronic Data Capture (REDCap) database [34]. Demographic information collected during the recruitment of caregivers and health care providers will be stored in an excel spreadsheet with participant names (and any other identifying information) removed and replaced with a descriptor and number (e.g. CG_01). Video recording data and field note data will be recorded directly onto password-protected laptop. Video files and field notes will then be securely transferred at the end of each recording day using a shared REDCap database that is stored on a health centre server. All caregiver demographic data, STAI and METER instrument data, chart audit data and caregiver follow-up survey data will be inputted directly into the secure, encrypted REDCap database stored on a health centre server. Only members of the research team will be granted access to the database. For data monitoring purposes, only the REDCap Project Administrator will be able to view all data from all sites. Users will be assigned to “Data Access Groups” (DAG) that will restrict their rights to viewing and entering data for their site alone. Within the DAG, user privileges will be designated to ensure research team members have only the minimum required rights to perform their duties.

### Data analysis

Video recordings will be analyzed using Observer XT, an event logging software. Data will be coded using two coding strategies. First, to determine whether essential content was included in the discharge communication, the videos will be scored yes/no as to whether they include the five discharge messages identified in a Delphi study [33]. Second, to analyze the primary outcome of provider-caregiver communication, recordings will be analyzed using the Roter Interaction Analysis System (RIAS) [35]. The RIAS coding system is designed to capture dynamic patient-provider communication and categorizes verbal communication behaviour into two broad categories, task-focused and emotion-focused, with further subcategorization based on their function (e.g., asks for understanding, gives information-medical condition). Each utterance will be tagged to an action (the person speaking) and a receiver (the person being spoken to). Codes will be summarized as rates (codes/min) or proportions (code x/total codes), providing an overall description of the types of clinician and caregiver communication behaviors observed during the encounter. In order to provide a context for interpretation of the RIAS data, we will also code the amount of time each clinician spends in the room with the patient, the number of interruptions in the dialog, and the overall length of time spent communicating. Non-verbal behaviours such as eye contact, body orientation (sitting or standing position), gestures (head nodding, hand movements), touch or any physical movement in the patient's room during the exchange of information (another child distracting parent, caregiver or healthcare provider looking at chart or phone) will also be coded. To assess reliability of coding, a second research assistant will code 30% of the available videos. Inter-rater reliability will be assessed using Pearson correlations and using intra-class correlations (ICC) to correct for dependency in the data. Disagreement in coding will be reviewed and incorporated into codebooks as needed.

Data from the caregiver intake forms, chart audit and follow-up surveys with caregivers will be analyzed using descriptive statistics. Caregiver recall will be estimated by calculating percentage agreement between self-report at follow up, data extracted from the chart and events observed during the video recording. Pearson correlation or Spearman’s rho correlation, depending on the normality of the data, will be used to analyze the bivariate association between caregiver recall and comprehension and caregiver demographics, child characteristics, verbal and non-verbal communication behaviours of healthcare providers and caregivers, and ED department characteristics during the visit. We will use logistic regression to identify potential demographic and visit characteristic confounders and multivariate logistic regression to examine association between verbal and non-verbal behaviours and parent recall and comprehension while adjusting for confounders.

### Recruitment and informed consent

All ED healthcare providers will be notified of the project by way of an informational letter distributed to the staff through an email distribution by a research assistant (RA). Clinicians will also be notified of the project and invited to consent during regularly scheduled staff meetings, orientation sessions, or teaching rounds. If interested in participating in the study, staff will then be asked to read the consent form, to contact the RA if they have any questions about the study, and to sign the consent form once all questions have been answered and they are in agreement with participating in the study. Ongoing verbal consent from all ED healthcare providers will be acquired prior to any collection of data (e.g., before video recording commences).

A list of all ED healthcare providers and trainees who do not wish to participate in the study will be kept. At the beginning of each week the RA will cross reference this list with the staffing schedule to determine which shifts include healthcare providers who did not wish to participate. These shifts will be designated as non-recruitment shifts. This will be done to ensure that providers who do not wish to participate in the study will not be identified to their colleagues. If a provider that has not consented enters the recording room, then recording will cease immediately.

Child and caregiver participants will be identified by an RA for inclusion based on the inclusion criteria of age, presenting illness, acuity, and presence of a caregiver. The RA will inform potential participants of their eligibility to participate in a study and will ask them if they are interested in getting more information. If they are interested, the RA will explain the study via the information letter and consent form, answering any questions they might have, and seek informed consent from the caregiver. If developmentally appropriate, the RA will also seek verbal assent from the child. Caregiver participants will receive a gift card upon completion of study participation to improve recruitment and retention.

## Discussion

While previous research has explored patient comprehension of ED discharge communication within the adult population using follow-up interviews and audio recording of communication, to our knowledge this is the first study of ED discharge communication to be conducted within the pediatric setting and using video observation methods [10, 36–38]. This novel research design will ensure that the “black box” of discharge communication is further elucidated through real-time observation. This reduces the limitations placed on previous research due to self-report. As well, this research will highlight the caregiver experience of discharge communication and will provide greater understanding of how caregiver recall and comprehension of discharge instructions is influenced by factors such as ED context or caregiver and provider communication behaviour.

We anticipate several challenges to this study design. Given the novel nature of video observation in a pediatric ED setting, it is possible that there might be some hesitancy on the part of ED healthcare providers to participate. To address this potential barrier, we have planned individual and group information sessions at each site to provide specific details about the video data management and analysis procedures. Participants will be informed that in recent studies using video recording of health encounters in a pediatric clinic setting, there was negligible evidence that video recording affected communication during the clinic visit [39]. This study is an important first step in elucidating the caregiver experience of discharge communication and will uncover the types of communication behaviours that may improve caregiver comprehension. Challenges may also occur as a result of having stationary video equipment in a patient room assessment room in the ED. This may necessitate changes to patient flow to ensure that eligible and consented patients/caregivers are placed in the assessment room with the cameras. We have attempted to minimize disruption by outfitting two patient assessment rooms with cameras. The RA will also work closely with the ED administrative staff to ensure patient care is not affected in the ED. The RA will also document episodes of provider-caregiver communication that may occur outside of the patient’s room. Another potential barrier that has been addressed is that of participant privacy. Several steps will be taken to ensure privacy, including transmitting video through a hardwire set up, disabling wireless internet access on the study laptop, installing a privacy screen on the study laptop, and ensuring that the RA is located in a position where the laptop screen is facing the wall.

This study design will address limitations identified in previous discharge communication research designs using only audio-recorded data [10, 38]. Video recording will provide an opportunity to capture important data such as temporality and sequence that might influence the communication process. Video recording will also provide a means to accurately record and code all possible distractions that occur during the interaction. Unlike previous studies that have utilized audio recording of interactions, video observation will allow for coding on non-verbal behaviours as well, allowing us to characterize other types of communications and behaviour that may influence caregiver comprehension and recall of discharge communication.

Given the importance of better characterizing discharge communication to identify potential barriers and enablers, we anticipate that the findings from this study will contribute to the development of more effective discharge communication policies and interventions. Additionally, as this study is the first-of-its-kind to use video observational data to inform intervention design in a pediatric ED setting, we anticipate our findings will make a important contribution to pediatric emergency care research and provide an important precedence for further use of video observation studies in this context. We plan to disseminate our findings to study participants through the distribution of a summary report, to health care professionals through established networks such as PERC and Translating Emergency Knowledge for Kids (TREKK), and to the broader community through publication in open-access journals and through presentation of results at professional conferences. The International Committee of Medical Journal Editors (ICMJE) criteria will be used to establish authorship for all resulting journal articles. Study findings will be reported following the STROBE guidelines.

In conclusion, this work has the potential for advancing the field of implementation science and intervention design. This study will be the first to analyze healthcare provider-caregiver communication in a pediatric ED setting using video observation. The inclusion of ED context, chart audit and caregiver follow-up data will contribute to developing a rich and comprehensive description of discharge communication to illuminate targets for change.

## Abbreviations

CTAS: Canadian Triage and Acuity Scale
ED: Emergency Department
ICC: Inter-class coefficient
ICMJE: The International Committee of Medical Journal Editors
METER: Medical Term Recognition Test
RA: Research Assistant
REDCap: Research Electronic Data Capture
RIAS: Roter Interaction Analysis System
